# Is the presence of keratinized mucosa associated with periimplant tissue health? A clinical cross-sectional analysis

**DOI:** 10.1186/s40729-015-0009-z

**Published:** 2015-04-29

**Authors:** Catharina Ladwein, Rainer Schmelzeisen, Katja Nelson, Tabea Viktoria Fluegge, Tobias Fretwurst

**Affiliations:** Department of Oral and Craniomaxillofacial Surgery, Center for Dental Medicine, University Medical Center Freiburg, Hugstetter Straße 55, D-79106 Freiburg, Germany

**Keywords:** Periimplantitis, Keratinized mucosa, Attached gingiva, Dental implants, Implantology

## Abstract

**Background:**

Long-term data concerning the impact of missing keratinized mucosa (KM) on periimplant tissue health are rare. The importance of KM for implant success remains unclear.

**Methods:**

Two hundred eleven patients with 967 dental implants were analyzed up to 15 years after implant placement. Implants were divided into two groups: no keratinized mucosa (NKM) and KM. Evaluated parameters were plaque index (mAPI), bleeding index (mSBI), bleeding on probing (BOP), probing depth (PD), width of KM, and radiographic vertical bone level.

**Results:**

mAPI, mSBI, and BOP were significantly higher for the NKM group. Implants of both groups showed no significant difference in PD and vertical bone level. Of the implants in the posterior regions (*n* = 261), 40.3% (regions 37 to 34, 44 to 47, 27 to 24, 17 to 14) showed NKM, whereas 30.4% of the implants in the anterior regions (regions 13 to 23; regions 33 to 43) presented NKM (*n* = 97).

**Conclusions:**

Results indicate that the presence of KM has a positive effect on periimplant tissue health, but does not seem to have an influence on the periimplant bone level.

## Background

Various factors influence the long-term success of dental implants are bone quality and quantity, oral hygiene, medical conditions, mechanical factors such as the surgical procedure, and the subsequent prosthetic treatment [1-6]. The importance of keratinized mucosa (KM) surrounding the implant as a barrier against microorganisms and subgingival plaque as a factor for long-term success is discussed [7,8]. KM includes the gingival margin and the mucogingival junction [9]. A width of ≥2 mm of masticatory mucosa with ≥1 mm of attached gingiva has been proposed as adequate for gingival health [10]. Periimplant soft tissue differs from periodontal tissue [11-14]. The mucosa adjacent to the implant has been described to consist a marginal layer of junctional epithelium with a height (length) of 2 mm and a more apical zone of connective tissue with a height (length) of about 1.5 mm [11,15]. Periimplant mucosa is akin to scar tissue, and its fibers are orientated parallel to the implant surface [12,16]. Animal and human studies showed that the early soft tissue response to plaque is similar around teeth and implants, although periimplant tissue is supposed to be less resistant [17-22].

Hitherto the influence of KM on periimplant health remains to be discussed controversial [8,23,24]. One clinical study investigating implants failed to show the influence of the existence of KM on periimplant soft tissue health [23]. Current studies with moderate examination periods up to 5 years reported that higher plaque accumulation and gingival inflammation were associated with the absence of KM [25,26]. This statement is supported by several systematic reviews [27-29]. Dental implants with less than 2 mm of KM seem to be more prone to recession and alveolar bone loss, measured between the crestal bone level and a reference point on the implant surface [26,30]. To determine the clinical periimplant status, defined parameters are available: implant mobility, radiographic bone level, probing depth (PD), plaque and gingival indices, bleeding on probing (BOP), and width of the KM [7,30].

The aim of the present study was to evaluate the relationship between the existence of KM and periimplant health regarding a larger collective in the long-term.

## Methods

This clinical cross-sectional study was conceived in accordance with the Helsinki Declaration of 1964, as revised in 2013. The study has been approved by the local ethical committee (University Medical Center Freiburg, no. 326_09).

### Population

Between February 1995 and December 2005, patients of the Clinic of Oral and Maxillofacial Surgery (Freiburg, Germany) who received endosseous dental implants (Tissue Level Standard Plus/Standard) of the Straumann-Dental Implant System (Straumann AG, Basel, Switzerland) were included in this monocentric clinical study. With the database PROMetheus Version III (KRZ, University medical center Freiburg), 620 patients with 2,667 dental implants were identified. Only those patients were evaluated who were available for follow-up investigation in 2009 to 2010. An evaluation of the incidence and reason for ‘lost of follow-up’ were not performed.

### Exclusion criteria

Exclusion criteria were diabetes, diseases of bone metabolism like osteoporosis, plasmozytoma, and osseous metastases, further malignant diseases in the head and neck region, bisphosphonat-, chemo-, or radiotherapy in the anamnesis, immunosuppressive diseases/therapies, pregnancy, and patient’s age <18 years.

### Clinical parameters and measurements

Patients and implant-related parameters including gender, age at implant insertion, time of implant insertion, region of insertion, implant length, and implant diameter were assessed. The clinical examination was performed three times, with independent time points by a single examiner. Please refer to Tables 1, 2, and 3 for the description of the evaluated parameters.

**Table 1:** **Evaluated implant parameters**

**Clinical**	**Radiological**
mAPI [41] (see Table 2)	Radiographic bone level (vertical bone defect)
mSBI [41] (see Table 3)	
PD in millimeters at four sites of the implant [42]	
Presence or absence of BOP	
Width of the KM in millimeters at the mid-facial aspects of the implants marked with iodide solution [10,42]	
Implant mobility for single-tooth restorations [43]

**Table 2:** **Assessment of plaque accumulation by the mAPI**

	**Assessment of plaque accumulation**
Score 0	No detection of plaque
Score 1	Plaque recognized by running a probe across the smooth marginal surface of the implant
Score 2	Plaque can be seen by the naked eye
Score 3	Abundance of soft matter

**Table 3:** **Assessment of bleeding tendency by the mSBI**

	**Assessment of bleeding tendency**
Score 0	No bleeding when a periodontal probe is passed along the gingival margin adjacent to the implant
Score 1	Isolated bleeding spots visible
Score 2	Blood forms a confluent red line on margin
Score 3	Heavy or profuse bleeding

Measurements were performed manually using a plastic periodontal probe (Plast-o-Probe®, Dentsply, York, PA, USA). All clinically measured distances were rounded to the nearest millimeter. Panoramic radiographs were used to assess the radiographic vertical bone level [31,32]. To account for dimensional distortions, the length of each implant was measured and correlated to the actual implant length. The vertical distance of the rough/smooth border of the implant to the first implant-bone contact was measured at the mesial and distal of each implant to define vertical bone level. The smooth-rough border is located either 2.8 mm (Tissue Level Standard) or 1.8 mm (Tissue Level Standard Plus) from the implant shoulder; this was regarded when radiologic measurements were performed. All examinations were performed on digital radiographs with a virtual measurement tool (SIDEXIS neXt Generation 2.4^©^, Sirona Dental Systems GmbH, Bensheim, Germany). Standardized radiographic examinations (orthopantomograms) were performed immediately after implant placement, before implant uncovery, in the first year every 6 months, and thereafter annually. The radiological analysis was performed by a single examiner (CL).

Case definition (Healthy, Mucositis, Periimplantitis) was performed according to the criteria of the seventh and eighth Workshop of the European Federation of Periodontology: ‘In absence of previous radiographic records, a threshold vertical distance of 2 mm from the expected marginal bone level following remodeling post-implant placement is recommended, provided peri-implant inflammation is evident’ [33]. If several implants were placed in a single patient, these were considered as individual implants, independent of each other.

### Statistical analysis

Implants were divided into two groups: no keratinized mucosa (NKM) = 0 mm of keratinized mucosa and KM > 0 mm of keratinized mucosa. All quantitative parameters are shown as mean ± standard deviation. Statistical analyses were performed with Mann-Whitney *U* test and the chi-squared test using the software program SPSS 18.0 (PASW® Statistics, SPSS Inc., IBM Company, Chicago, IL, USA). A *p* value < 0.05 was considered significant.

## Results

Two hundred eleven patients (97 = male, 114 = female) with 967 dental implants were available for follow-up examination. The mean observation period was 7.78 years ± 1.92 (4 to 15 years); the mean age at implant insertion was 54.63 years ± 13.58 (maximum 78 years).

Two hundred seventy-five implants were placed in the posterior regions of the lower jaw (34 to 37, 44 to 47), and 143 implants were placed in the anterior region of the lower jaw (33 to 43). In the maxilla, 373 implants were placed in the posterior regions (14 to 17, 24 to 27) and 176 implants were inserted in regions 13 to 23. Three hundred fifty-eight implants (37.02 %) did show NKM and 609 implants (62.98 %) showed KM. Of the implants in the posterior regions, 40.3% (*n* = 261) did not present KM, whereas 59.7% (*n* = 387) of posterior implants had KM (see Table 4). In the anterior regions, 30.4% (*n* = 97) of the implants were classified NKM and 59.7% (*n* = 222) were classified KM.

**Table 4:** **Distribution of presence of keratinized/non-keratinized gingiva with regard to the region**

**Region**	**Number of implants**	**NKM**	**KM**
		***n*** **(%)**	***n*** **(%)**
Posterior region maxilla	373	134 (13.9)	239 (24.7)
Anterior region maxilla	176	54 (5.6)	122 (12.6)
Posterior region mandible	275	127 (13.1)	148 (15)
Anterior region mandible	143	43 (4.5)	100 (10.3)

The mean width of KM was 1.87 ± 1.82 mm. Considering plaque index (mAPI), bleeding index (mSBI), and BOP implants with NKM showed significantly more plaque accumulation and bleeding than implants with KM (see Table 5). PD and vertical bone defect were not significantly different between both groups (PD mesial 3.78 ± 1.57 mm (NKM) vs. 3.61 ± 1.48 mm (KM), *p* = 0.28) (see Table 6).

**Table 5:** **Results for mAPI, mSBI, and BOP for implants with either keratinized mucosa or no keratinized mucosa**

		**NKM (%)**	**KM (%)**	***p*** **value**
mAPI	Score 0	24.3	32.8	<0.05
	Score 1	19.0	36.1	
	Score 2	23.9	18.1	
	Score 3	32.7	12.9	
mSBI	Score 0	32.9	46.3	<0.05
	Score 1	20.8	28.4	
	Score 2	31.1	18.5	
	Score 3	15.2	6.8	
BOP	Distal	46.1	35.9	<0.05
	Buccal	43.7	32.1	<0.05
	Mesial	57.7	50.6	0.55

**Table 6:** **PD and bone level for implants regarding the soft tissue condition**

	**NKM**	**KM**	
	**Mean SD**	**Mean SD**	***p*** **value**
	**(mm)**	**(mm)**	
PD distal	3.3 ± 1.4	3.5 ± 1.5	0.21
PD buccal	2.9 ± 1.3	2.9 ± 1.3	0.81
PD mesial	3.8 ± 1.6	3.6 ± 1.5	0.28
Vertical bone defect distal	0.8 ± 1.4	0.7 ± 1.2	0.70
Vertical bone defect mesial	0.9 ± 1.2	0.8 ± 1.3	0.31

## Discussion

Multiple investigators discussed the necessity of KM around dental implants [28]. But still, there is a lack of evidence whether KM has an impact on periimplant tissue health and periimplant bone level, respectively [27,29]. The main reason for this limitation is the heterogeneity in study designs and data extraction; consecutively, there is a lack of a synoptic statistical analysis [34]. The present study examined the aforementioned issue in a large collective in the long-term. According to a recent review comparing 19 relevant publications out of 217 articles, only 1 study has a comparable, slightly larger, collective with a similar observation period (>10 years) [34,35]. Roos-Jansaker et al. demonstrated with a univariate and multivariate analyses that the presence of KM is associated with a mucositis [35].

The influence of KM on oral plaque accumulation and hygiene measures is discussed controversially in literature. In the present study, patients with NKM show significantly more plaque accumulation than patients with KM. Human studies with substantially smaller cohorts confirm these results and reported even higher plaque scores at implant sites in the absence of KM [7,25,26,30,36,37] On the other hand, several longitudinal studies revealed no significant association between higher plaque scores and KM level [11,23,38,39].

A similar ambivalence exists for the bleeding after probing parameter in literature [34]. The present study shows significantly higher values of BOP and mSBI in the absence of KM. KM is supposed to be a physical barrier, and the absence of it seems to provide easier apical migration of inflammation [28,30]. KM has been assumed to prevent mechanical damage evoked by toothbrushing and/or masticatory forces [20,39].

In this study, only one implant type (Straumann, Tissue Level) with a polished neck was examined. Therefore, the acquired data may not represent different implant designs and emergence profiles. Some authors established studies with a range of different implant surfaces like sandblasted with large grit and acid-etched surfaces or anodized surfaces. Other authors give no description of the implant surface conditions [7,39]. The implant type and material may influence the periimplant parameters especially the bone level. It is discussed whether machined surfaces are more favorable for periimplant soft tissue parameters [40]. But different implant surface conditions may alter the effect on KM [28,40]. To identify influencing factors like implant surface or occlusal overload, longitudinal prospective studies with a standardized protocol are needed.

In the present study, the mean width of KM was 1.87 ± 1.82 mm. Thus, our results suggest that even smaller amounts of KM may be sufficient for periimplant health, in contrast to the values claimed by other authors [7,10]. According to our state of knowledge, no data exist on the amount of KM remaining after the removal of a tooth with consideration of the anatomical region. Our data reveal no difference in the amount of KM in certain anatomical regions of the maxilla and mandible.

There was no significant difference in PD and vertical bone defect between both groups. A 4-year prospective longitudinal study confirms these results and showed no significant difference in the proximal periimplant bone level between implants with KM or NKM, respectively [36]. Cross-sectional studies reported an impact of missing KM on the mean loss of alveolar bone around implants [30,39]. Large, randomized, and long-term multicenter studies with regard to the different implant surfaces and materials are necessary to investigate further association between the existence of KM and periimplant bone level.

## Conclusions

The present findings indicate a correlation between periimplant soft tissue health and the presence of KM. Dental implants lacking KM showed significantly more plaque accumulation and BOP than implants with a zone of KM. The presence of KM did not have a significant influence on the vertical periimplant bone level.

## References

[1] Belibasakis GN (2014). Microbiological and immuno-pathological aspects of peri-implant diseases. Arch Oral Biol.

[2] Cappiello M, Luongo R, Di Iorio D, Bugea C, Cocchetto R, Celletti R (2008). Evaluation of peri-implant bone loss around platform-switched implants. Int J Periodontics Restorative Dent.

[3] Cehreli M, Sahin S, Akça K (2004). Role of mechanical environment and implant design on bone tissue differentiation: current knowledge and future contexts. J Dent.

[4] Karthik K, Sivakumar S, Thangaswamy V (2013). Evaluation of implant success: a review of past and present concepts. J Pharm Bioallied Sci.

[5] Peñarrocha-Diago MA, Flichy-Fernández AJ, Alonso-González R, Peñarrocha-Oltra D, Balaguer-Martínez J, Peñarrocha-Diago M (2013). Influence of implant neck design and implant-abutment connection type on peri-implant health. Radiological study. Clin Oral Implants Res.

[6] Steigenga JT, Al-Shammari KF, Nociti FH, Misch CE, Wang HL (2003). Dental implant design and its relationship to long-term implant success. Implant Dent.

[7] Adibrad M, Shahabuei M, Sahabi M (2009). Significance of the width of keratinized mucosa on the health status of the supporting tissue around implants supporting overdentures. J Oral Implantol.

[8] Wennström JL, Bengazi F, Lekholm U (1994). The influence of the masticatory mucosa on the peri-implant soft tissue condition. Clin Oral Implants Res.

[9] Orban B (1948). Clinical and histologic study of the surface characteristics of the gingiva. Oral Surg Oral Med Oral Pathol.

[10] Lang NP, Löe H (1972). The relationship between the width of keratinized gingiva and gingival health. J Periodontol.

[11] Abrahamsson I, Berglundh T, Wennström J, Lindhe J (1996). The peri-implant hard and soft tissues at different implant systems. A comparative study in the dog. Clin Oral Implants Res.

[12] Berglundh T, Lindhe J, Ericsson I, Marinello CP, Liljenberg B, Thomsen P (1991). The soft tissue barrier at implants and teeth. Clin Oral Implants Res.

[13] Berglundh T, Lindhe J, Jonsson K, Ericsson I (1994). The topography of the vascular systems in the periodontal and peri-implant tissues in the dog. J Clin Periodontol.

[14] Ericsson I, Lindhe J (1993). Probing depth at implants and teeth. An experimental study in the dog. J Clin Periodontol.

[15] Berglundh T, Lindhe J (1996). Dimension of the periimplant mucosa. Biological width revisited. J Clin Periodontol.

[16] Buser D, Weber HP, Donath K, Fiorellini JP, Paquette DW, Williams RC (1992). Soft tissue reactions to non-submerged unloaded titanium implants in beagle dogs. J Periodontol.

[17] Ericsson I, Berglundh T, Marinello C, Liljenberg B, Lindhe J (1992). Long-standing plaque and gingivitis at implants and teeth in the dog. Clin Oral Implants Res.

[18] Leonhardt A, Berglundh T, Ericsson I, Dahlén G (1992). Putative periodontal pathogens on titanium implants and teeth in experimental gingivitis and periodontitis in beagle dogs. Clin Oral Implants Res.

[19] Schou S, Holmstrup P, Stoltze K, Hjørting-Hansen E, Fiehn NE, Skovgaard LT (2002). Probing around implants and teeth with healthy or inflamed peri-implant mucosa/gingiva. A histologic comparison in cynomolgus monkeys (Macaca fascicularis). Clin Oral Implants Res.

[20] Schou S, Holmstrup P, Hjørting-Hansen E, Lang NP (1992). Plaque-induced marginal tissue reactions of osseointegrated oral implants: a review of the literature. Clin Oral Implants Res.

[21] Zitzmann NU, Berglundh T, Marinello CP, Lindhe J (2001). Experimental peri-implant mucositis in man. J Clin Periodontol.

[22] Pontoriero R, Tonelli MP, Carnevale G, Mombelli A, Nyman SR, Lang NP (1994). Experimentally induced peri-implant mucositis. A clinical study in humans. Clin Oral Implants Res.

[23] Adell R, Lekholm U, Rockler B, Brånemark PI, Lindhe J, Eriksson B (1986). Marginal tissue reactions at osseointegrated titanium fixtures (I). A 3-year longitudinal prospective study. Int J Oral Maxillofac Surg.

[24] Mericske-Stern R, Steinlin Schaffner T, Marti P, Geering AH (1994). Peri-implant mucosal aspects of ITI implants supporting overdentures. A five-year longitudinal study. Clin Oral Implants Res.

[25] Chung DM, Oh TJ, Shotwell JL, Misch CE, Wang HL (2006). Significance of keratinized mucosa in maintenance of dental implants with different surfaces. J Periodontol.

[26] Schrott AR, Jimenez M, Hwang JW, Fiorellini J, Weber HP (2009). Five-year evaluation of the influence of keratinized mucosa on peri-implant soft-tissue health and stability around implants supporting full-arch mandibular fixed prostheses. Clin Oral Implants Res.

[27] Gobbato L, Avila-Ortiz G, Sohrabi K, Wang CW, Karimbux N (2013). The effect of keratinized mucosa width on peri-implant health: a systematic review. Int J Oral Maxillofac Implants.

[28] Brito C, Tenenbaum HC, Wong BK, Schmitt C, Nogueira-Filho G (2014). Is keratinized mucosa indispensable to maintain peri-implant health? A systematic review of the literature. J Biomed Mater Res B Appl Biomater.

[29] Lin GH, Chan HL, Wang HL (2013). The significance of keratinized mucosa on implant health: a systematic review. J Periodontol.

[30] Bouri A, Bissada N, Al-Zahrani MS, Faddoul F, Nouneh I (2008). Width of keratinized gingiva and the health status of the supporting tissues around dental implants. Int J Oral Maxillofac Implants.

[31] Kullman L, Al-Asfour A, Zetterqvist L, Andersson L (2007). Comparison of radiographic bone height assessments in panoramic and intraoral radiographs of implant patients. Int J Oral Maxillofac Implants.

[32] Zechner W, Watzak G, Gahleitner A, Busenlechner D, Tepper G, Watzek G (2003). Rotational panoramic versus intraoral rectangular radiographs for evaluation of peri-implant bone loss in the anterior atrophic mandible. Int J Oral Maxillofac Implants.

[33] Sanz M, Chapple IL, Working Group 4 of the VIII European Workshop on Periodontology (2012). Clinical research on peri-implant diseases: consensus report of Working Group 4. J Clin Periodontol.

[34] Wennström JL, Derks J (2012). Is there a need for keratinized mucosa around implants to maintain health and tissue stability?. Clin Oral Implants Res.

[35] Roos-Jansåker AM, Renvert H, Lindahl C, Renvert S (2006). Nine- to fourteen-year follow-up of implant treatment. Part III: factors associated with peri-implant lesions.. J Clin Periodontol.

[36] Crespi R, Capparè P, Gherlone E (2010). A 4-year evaluation of the peri-implant parameters of immediately loaded implants placed in fresh extraction sockets. J Periodontol.

[37] Boynueğri D, Nemli SK, Kasko YA (2013). Significance of keratinized mucosa around dental implants: a prospective comparative study. Clin Oral Implants Res.

[38] Heckmann SM, Schrott A, Graef F, Wichmann MG, Weber HP (2004). Mandibular two-implant telescopic overdentures. Clin Oral Implants Res.

[39] Kim BS, Kim YK, Yun PY, Yi YJ, Lee HJ, Kim SG (2009). Evaluation of peri-implant tissue response according to the presence of keratinized mucosa. Oral Surg Oral Med Oral Pathol Oral Radiol Endod.

[40] Lee S, Goh BT, Wolke J, Tideman H, Stoelinga P, Jansen J (2010). Soft tissue adaptation to modified titanium surfaces. J Biomed Mater Res A.

[41] Mombelli A, van Oosten MA, Schurch E, Land NP (1987). The microbiota associated with successful or failing osseointegrated titanium implants. Oral Microbiol Immunol.

[42] Buser D, Weber HP, Brägger U (1990). The treatment of partially edentulous patients with ITI hollow-screw implants: presurgical evaluation and surgical procedures. Int J Oral Maxillofac Implants.

[43] Lindhe J, Nyman S (1977). The role of occlusion in periodontal disease and the biological rationale for splinting in treatment of periodontitis. Oral Sci Rev.

